# Anaerobic and Aerobic Performances in Elite Basketball Players

**DOI:** 10.2478/hukin-2014-0068

**Published:** 2014-10-10

**Authors:** Gustavo Gomes de Araujo, Fúlvia de Barros Manchado-Gobatto, Marcelo Papoti, Bruno Henrique Ferreira Camargo, Claudio Alexandre Gobatto

**Affiliations:** 1 Laboratory of Sports Applied Physiology, University of Campinas (UNICAMP).. Faculty of Applied Sciences (FCA), Limeira, SP – Brazil; 2 Sports Science Research Group, Federal University of Alagoas (UFAL).CEDU/Physical Education and PPGNUT Campus A.C. Simões, Maceió, AL – Brazil.; 3 University of São Paulo (USP) School of Physical Education and Sports (EFERP), Monte Alegre, Ribeirão Preto, SP – Brazil.

**Keywords:** lactate minimum, sprint, mathematical model, polynomial adjust

## Abstract

The purpose of this study was to propose a specific lactate minimum test for elite basketball players considering the: Running Anaerobic Sprint Test (RAST) as a hyperlactatemia inductor, short distances (specific distance, 20 m) during progressive intensity and mathematical analysis to interpret aerobic and anaerobic variables. The basketball players were assigned to four groups: All positions (n=26), Guard (n= 7), Forward (n=11) and Center (n=8). The hyperlactatemia elevation (RAST) method consisted of 6 maximum sprints over 35 m separated by 10 s of recovery. The progressive phase of the lactate minimum test consisted of 5 stages controlled by an electronic metronome (8.0, 9.0, 10.0, 11.0 and 12.0 km/h) over a 20 m distance. The RAST variables and the lactate values were analyzed using visual and mathematical models. The intensity of the lactate minimum test, determined by a visual method, reduced in relation to polynomial fits (2nd degree) for the Small Forward positions and General groups. The Power and Fatigue Index values, determined by both methods, visual and 3rd degree polynomial, were not significantly different between the groups. In conclusion, the RAST is an excellent hyperlactatemia inductor and the progressive intensity of lactate minimum test using short distances (20 m) can be specifically used to evaluate the aerobic capacity of basketball players. In addition, no differences were observed between the visual and polynomial methods for RAST variables, but lactate minimum intensity was influenced by the method of analysis.

## Introduction

Basketball is considered an intermittent high-intensity sport that requires mainly anaerobic metabolism (Castagna et al., 2009; Hoffman et al., 1999). It is known that the anaerobic contribution in basketball is important for tactical moves (i.e., defensive/offensive transitions) and technical actions such as shooting, jumping, blocking, passing, lay-ups and other technical movements (Castagna et al., 2010; Delextrat et al., 2008; Hoffman et al., 1999). However, the duration of a basketball game (40–48 min) requires a high level of aerobic metabolism to enhance the resynthesis of creatine phosphate, lactate clearance from active muscle and removal of accumulated intracellular inorganic phosphate (Glaister, 2005). In this context, it seems clear that the physical fitness of basketball players and game performance can be influenced by both aerobic and anaerobic metabolism (Montgomery et al., 2010; Narazaki et al., 2009). Thus, these interactions between aerobic and anaerobic metabolism should also be considered in the evaluation and training prescription. Although there are many evaluation protocols to identify the aerobic and anaerobic performances in athletes (Balčiūnas et al., 2006; Ben Abdelkrim et al., 2010; Delextrat et al., 2008), few protocols have been applied to specifically evaluate these conditions in acyclic sports (Castagna et al., 2010). For this reason, the elaboration of specific tests to evaluate aerobic and anaerobic performance in basketball players can be important for researchers, coaches and athletes to optimize training programs and performance.

The lactate minimum test (LacMin) has been widely employed to predict the maximal lactate steady state intensity in multiple sports (Faude et al., 2009; Knoepfli-Lenzin and Boutellierm, 2011; Tegtbur et al., 1993). Differently from other protocols, the LacMin enables to assess the aerobic and anaerobic variables in a single session since it consists of an incremental exercise after hyperlactatemia induction (i.e. specific protocol for anaerobic performance) to determine the point in which the rate of lactate production is the same as the rate of lactate removal (Castagna et al., 2008; Johnson et al., 2009; Knoepfli-Lenzi and Boutellierm, 2011). Although there are several studies regarding the LacMin protocol, no studies have explored the LacMin test for basketball players using field conditions (Johnson et al., 2009; Ribeiro et al., 2009; Sotero et al., 2009; Smith et al., 2002). Adapting the LacMin test may be important in precisely assessing the aerobic and anaerobic performances on the court (Jones and Dousty, 1998; Tegtbur et al., 1993; Knoepfli-Lenzi and Boutellierm, 2011).

The running anaerobic sprint test (RAST) is a protocol used in basketball and field sports to evaluate the anaerobic power and the fatigue index (Balčiūnas et al., 2006). Despite the RAST has been widely used by coaches and athletes, few studies have investigated the applicability of this protocol in sport (Balčiūnas et al., 2006; Zacharogiannis et al., 2004; Zagatto et al., 2009). Studying the RAST protocol in elite basketball players may be important either to improve the scientific knowledge about the running anaerobic performance or to establish the reference values for basketball. The variables obtained during the RAST are evaluated using simple equations (Balčiūnas et al., 2006; Zacharogiannis et al., 2004; Zagatto et al., 2009), but there are no studies in the literature that use rigorous criteria (i.e., mathematical function) to calculate these variables in basketball players. This approach in acyclic sports enables to standardize the RAST determination using a mathematical criterion and verify the differences between non-mathematical and mathematical methods with practical implications.

In this context, the study aimed to propose a specific lactate minimum test for elite basketball players considering the RAST as a hyperlactatemia inductor and short distance (specific distance, 20 m) during progressive intensity, and to investigate the differences between mathematical and non-mathematical methods on LacMin and RAST variables. It was hypothesized that aerobic capacity of basketball players could be evaluated using a specific protocol of lactate minimum and mathematical interpretations would not differ from visual inspection to determine the aerobic and anaerobic index.

## Material and Methods

### Participants

A total of 26 elite Brazilian basketball players (21±5.0 yrs, body mass 96.6±14.8 kg, body height 1.95 ± 0.07 m and body mass index 24.9±2.6 kg/m^2^) were evaluated during the initial phase of the competitive period. The sample included 50% of Brazilian National Team Players and 50% of the Elite National League. The sample was divided according to specific positions of basketball: Guard (n=7, 21±6 yrs, body mass 83.3±5.2 kg, body height 1.87±0.03 m and body mass index 23.8±1.6 kg/m^2^), Forward (n=11, 22±4 yrs, body mass 94.5±4.8 kg, body height 1.97±0.05 m and body mass index 24.3±1.1 kg/m^2^) and Center (n=8, 21±3 yrs, body mass 109.4±19.1 kg, body height 2.01±0.04 m and body mass index 26.8±3.9 kg/m^2^). All players gave their informed consent to participate and the study protocol was approved by an independent review board in accordance to the ethics committee of the Campinas State University (UNICAMP), Brazil. Procedures

### Running Anaerobic Sprint Test

The RAST was used to determine the fatigue index (FI) and power: maximum (Pmax), average (Pavr) and minimum (Pmin). The RAST was applied after a warm-up (10 min) and was performed on a track. The test consisted of six maximum sprints over 35 m with an interval of 10s between sprints.

The velocity, acceleration, force and power were determined by the following equations: 1) Velocity (m/s)= Distance/Time; 2) Acceleration (m/s^2^)= Velocity/Time; 3) Force (kg^*^m^*^s^−2^)= Weight ^*^ Acceleration; 4) Power (Watts) = Force ^*^ Speed.

The determination of non-mathematical Pmax, Pavr, Pmin and FI were calculated: Pmax= maximum power value among the six sprints; Pavr= ∑ of 6 power values/6; Pmin= minimum power value among the six sprints; Fatigue Index 1 (Watts/kg/s) = (maximum power - minimum power)/ ∑ of six sprints/kg; and Fatigue Index 2 (%) = [(maximum power - minimum power)/maximum power] × 100.

**Figure 1:**
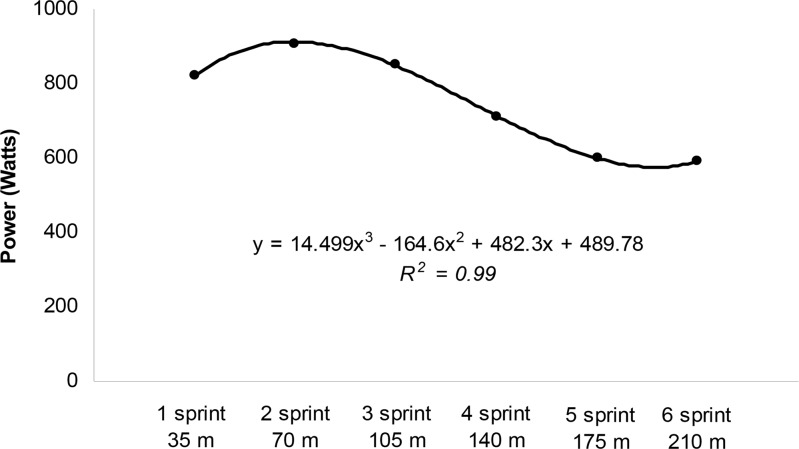
Example of third degree polynomial fit curves (Power of each bout vs. distances) to mathematically determine (deriving the third degree equation and calculating the x max and x min) the RAST variables (Pmax, Pmin, Pavr and FI) and distances (x values) of the Pmax and Pmin

The mathematical power values and FI were calculated using third degree polynomial fit curves (Figure 1). From the cubic equation (y= ax^3^-bx^2^ + cx + d), we calculated Pmax, Pavr, Pmin and FI (1 and 2). Deriving the third degree equation (0= ax^2^ + bx + c), we obtained the maximal and minimum *x* values by: 1) Δ= b^2^ - 4^*^a^*^c; 2) *x* max= -b + √Δ/2^*^a; 3) *x* min= -b - √Δ/2^*^a.

The *x* max and *x* min values were replaced in the cubic equation (y= ax^3^- bx^2^ + cx + d) to determine *y* max (Pmax) and *y* min (Pmin). The Pavr was determined by replacing the *x* values of cubic equation by: 1, 2, 3, 4, 5 and 6 (Figure 1). The R^2^ lower than 0.80 was considered insufficient to third degree polynomial fit. After the RAST, blood samples were collected at 5, 7 and 9 min for lactate peak (Lac Peak) determination in order to start the incremental phase (Smith et al., 2002).

### Aerobic Performance (LacMin)

The lactate minimum test (LacMin) was used to determine aerobic performance. This test consists of a first phase of hyperlactatemia induction (RAST) followed by increases in running intensity. The progressive phase consists of 5 stages (3 min each) with velocities of 8.0, 9.0, 10.0, 11.0 and 12.0 km/h controlled by an electronic metronome over a 20 m distance. The stages were separated by 30 s for blood collection.

### Lactate Minimum Test Parameters

**Figure 2:**
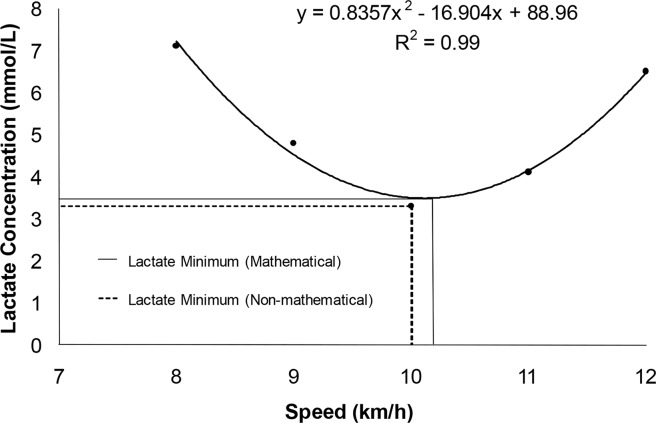
Example of a lactate minimum test of a participant. Hyperlactatemia was induced by the RAST, and the incremental phase was achieved with speeds of 8.0, 9.0, 10.0, 11.0 and 12.0 km/h. The stages were separated by 30 s for blood collection and lactatemia determination. Lactate Minimum Equation: The lactate vs. speed was fit by a second order polynomial. The minimum lactate intensity was obtained considering y=0, while the lactate minimum concentration was determined by replacing the LacMin intensity values in x equation. Lactate Minimum Visual: The lactate minimum intensity and concentration was obtained by visual inspection

The LacMin values were obtained from the derived equal zero of the second order polynomial fit and by visual inspection for the lowest lactate value of the “U-shaped” curve of blood lactate concentration versus velocity in the incremental phase of the LacMin test. To calculate the LacMin concentration, the LacMin intensity calculated with the second order polynomial fit values in *x* were replaced from the second order polynomial equation (de Araujo et al., 2007). In addition, the LacMin concentration and intensity were obtained by visual inspection (Figure 2). The Δ Lactate was calculated by LacPeak - LacMin concentration. From the equation y=ax^2^ – bx + c, we considered y=0 and derived the 2^nd^ degree equation: 1) y=ax^2^ – bx *+ c;* 2) 0=ax^2^ – bx; 3) 0=2^*^ax – b; 4) ax=b; 5) x= b/a.

### Blood Sample and Analysis

Blood samples (25 μL) were collected after the progressive stages, 8.0, 9.0, 10.0, 11.0 and 12.0 km/h, from the earlobe using heparinized capillary tubes. Samples were transferred to 1.5 mL microcentrifuge tubes containing 50 μL of sodium fluoride (1%). The blood lactate was analyzed with a specific analyzer (YSI 1500 Sport^®^).

### Statistical Analysis

The dependent variables collected throughout the research were subjected to the normality test using the W test of Shapiro-Wilk. The Student’s t-test was applied for independent variables for values (RAST and LacMin) obtained between mathematical and non-mathematical analysis. For variables (RAST and LacMin) obtained among groups (all positions, guard, forward and center) one-way analysis (ANOVA) was applied. When a significant interaction effect was found, a Tukey HSD post hoc test was used to identify where the difference existed among groups (Statistica 7.0^®^ Statsoft, Tulsa, OK). The product-moment (Correlation Matrices) test was applied in all variables of the RAST (visual and mathematical) and LacMin (visual and mathematical). The statistical significance was set at p<0.05, and all data are presented as mean ± *standard error* (SE). The effect size (ES) was calculated for all variables between nonmathematical and mathematical analyses. The thresholds for small, moderate, and large effects were 0.20, 0.50, and 0.80, respectively. The ES was determined by the formula: (mean1 - mean2)/pooled standard deviation.

## Results

The absolute and relative power values and FI were estimated by the mathematical and non-mathematical/visual inspection (Table 1). No differences were visualized between the methods (mathematical and non-mathematical) as well as among the positions for all variables of the RAST (Table 1). Also, the ES between mathematical and visual inspection for Guard players were: Pmax (W)= 0.38; Pmax (W/kg/s)=0.36; Pavr (W)= 0.35; Pavr (W/kg/s)= 0.32; Pmin (W)= 0.28; Pmin (W/kg/s)= 0.17; IF (%)= 0.14; IF (W/kg)= 0.32. The ES for Forward, Center and pooled positions were considered small (0.01 – 0.26) between the methods for all RAST variables.

The coefficients of determination in the third degree polynomial (R^2^) ranged from 0.80 to 0.99 (All positions= 0.92±0.01; Guard=0.90±0.01; Forward=0.88±0.01; Center=0.96±0.01).

The mean of LacPeak, LacMin concentration, LacMin intensity and Δ Lactate are shown in Table 2. By the values of LacPeak, the RAST proved be an appropriate protocol for hyperlactatemia induction in the lactate minimum test (LacMin). The intensity of LacMin measured by visual inspection was lower than mathematical polynomial fit for Forward (ES=0.50) and All positions (ES=0.47). However, for Guard (ES=0.51) and Center (ES=0.42), the visual determination of the LacMin was not significantly different from mathematical determination (Table 2).

The LacMin (mmol/L) and Δ Lactate (mmol/L) evaluated by mathematical and nonmathematical methods showed a small (0.23) and moderate (0.52) ES for the Center group, respectively. However, the ES values for Forward, Guard and All positions were considered smaller (0.05 – 0.17) than Center values for these variables.

**Figure 3:**
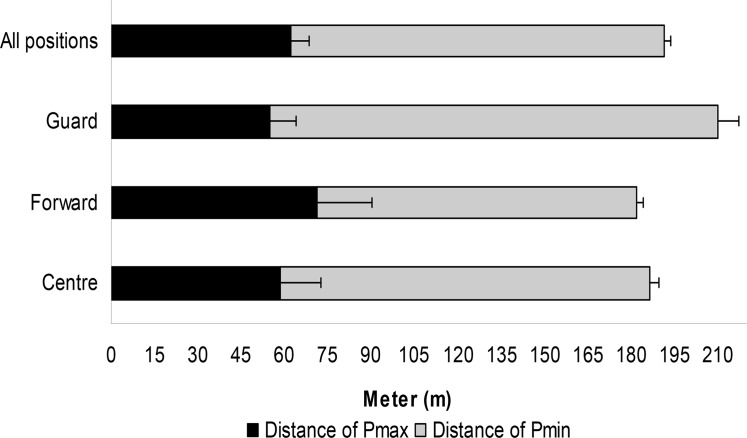
The third degree equation was derived by the maximal and minimum x values: 1) Δ= b^2^ - 4^*^a^*^c; 2) x max= -b + √Δ/2^*^a; 3) x min= -b - √Δ/2^*^a. The maximal and minimum x corresponded with the Pmax and Pmin distance (m)

The distance (m) to reach the Pmax and Pmin did not differ among the groups (Figure 3).

The correlation values obtained by mathematical analysis were higher (Pmax (W) *vs* FI (%)= 0.72; Pmax (W/kg) *vs* FI (W/kg/s)= 0.96; Pavr (W/kg) *vs* FI (W/kg/s)= 0.85) than the visual determination of RAST values (Pmax (W) *vs* FI (%)= 0.48; Pmax (W/kg) *vs* FI (W/kg/s)= 0.57; Pavr (W/kg) *vs* FI (W/kg/s)= 0.50).

The LacMin was significantly correlated with the FI, Pmax and Pavr determined by mathematical methods: Guard: FI (%) *vs* LacMin (km/h)= 0.99; All positions: Pmax (W/kg) *vs* LacMin (km/h)= 0.82; Pavr (W/kg) *vs* LacMin (km/h)= 0.80. Also, the correlation obtained by polynomial fit was higher than the visually-determined values for: Guard (FI (%) *vs* LacMin (km/h)= 0.21) and All players (Pmax (W/kg) *vs* LacMin (km/h)= 0.38; Pavr (W/kg) *vs* LacMin (km/h)= 0.53).

**Figure 4:**
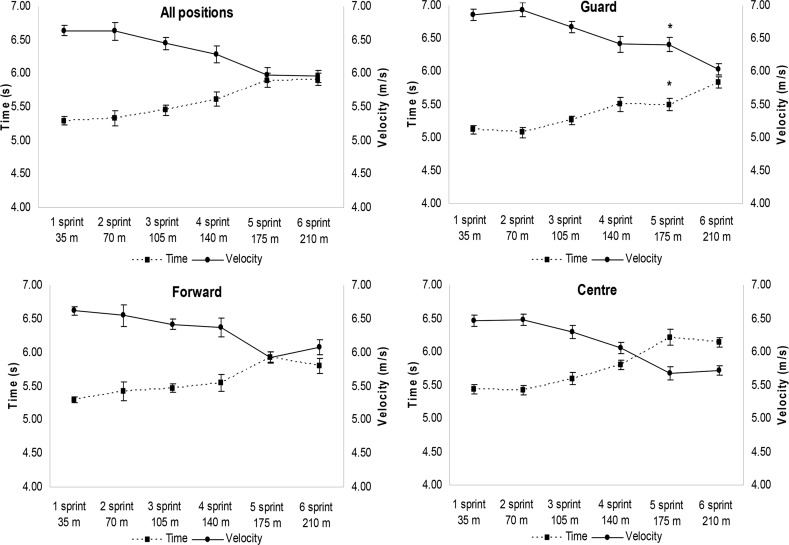
Mean ± Standard Error in Time (s) and Velocity (m/s) during six sprints for the different groups ^*^ Different in relation to the Center Group in the same period (p<0.05)

**Figure 5:**
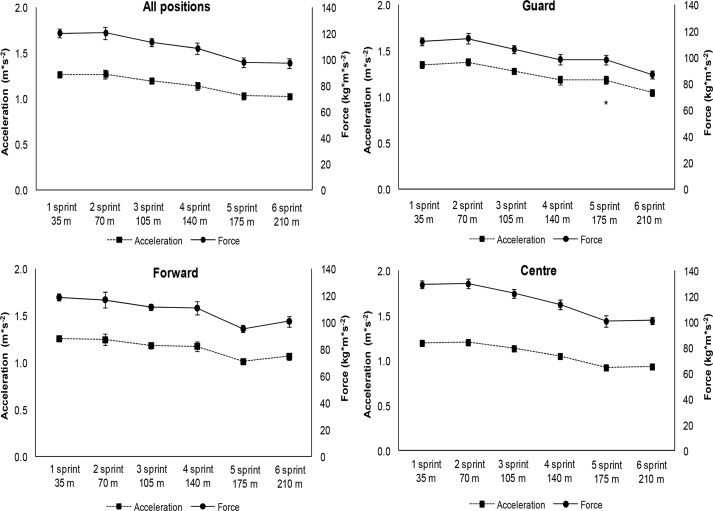
Mean ± Standard Error in Acceleration (m^*^s^−2^) and Force (kg^*^m^*^s^−2^) during six sprints for the different groups ^*^ Different in relation to the Center Group in the same period (p<0.05)

The time, velocity, acceleration and force were determined only by non-mathematical methods. These values are shown in Figures 4 and 5 for all groups. Differences between groups were observed during the fifth sprint between Guard and Center for the following parameters: time, velocity and acceleration (Figures 4 and 5).

## Discussion

In this study, the lactate minimum test was adapted for basketball players using the RAST to induce hyperlactatemia and a specific bidirectional test of 20 m during the incremental phase. Our data provided reference values of aerobic and anaerobic capacities for basketball players and standardized a single and specific protocol for team sports.

According to our results, the lactate kinetic (U-shaped) during the incremental phase complied with the theory proposed by Tegtbur et al. (1993). Fittings of curves that disabled the second order polynomial analysis, as well as values of R2 lower than 0.80, were considered insufficient for the assurance of the method (deAraujo et al., 2007). However, the regression coefficients obtained by polynomial adjust (2nd degree) for all players were high (R2 between 0.88–0.96). Thus, the polynomial adjust was important to establish a mathematical criteria to determine the maximal exercise load at which the lactate production rate was the same as the lactate removal rate (Ribeiro et al., 2003; Simões et al., 2002; Knoepfli-Lenzi and Boutellierm, 2011). The mathematical values of LacMin (km/h) were lower than those determined by visual inspection in the Forwards and All positions. Therefore, for in the Guards and Centers no differences were observed, but showed a moderate ES (0.51 and 0.42, respectively).

Several researchers compared the LacMin intensity obtained by non-mathematical and mathematical methods and did not find differences between the analysis (de Araujo et al., 2007; Ribeiro et al., 2009; Sotero et al., 2009; Simões et al., 2002). Ribeiro et al. (2003) compared LacMin intensity of swimmers determined by visual inspection and Spline function in stages of 200 m and 300 m crawl swimming. These authors did not observe differences between mathematical and non-mathematical methods. Simões et al. (2002) did not find differences between LacMin intensity using polynomial fit curves with 3 or 6 points in comparison to a visual inspection in cyclists. Pardono et al. (2008) determined the LacMin visually and through a quadratic polynomial function in cyclists and compared it with the maximal lactate steady-state workload (MLSSw). These authors did not find significant changes in aerobic capacity among the protocols (LacMin visual, LacMin mathematic and MLSSw). On the other hand, our data showed that the LacMin values obtained by the mathematical method reduced by 4, 3, 5 and 5% for All positions, Guards, Forwards and Centers, respectively, in relation to visual inspection. By the interpretation of all set results, the mathematical adjustment was considered an accurate process to determine the LacMin in relation to visual inspection (de Araujo et al., 2007; Simões et al., 2009). Therefore, further investigation is necessary to propose the MLSS in short distances (20 m) to compare with specific LacMin (Knoepfli-Lenzi and Boutellierm, 2011).

The bidirectional test using distance of 20 m, simulates the action in basketball games, but the constant accelerations and decelerations promote protocol dependence in comparison to continuous velocities (Castagna et al., 2010). The results in this study showed that the aerobic capacity determined by LacMin was lower than other multiple sprint sports evaluated by a continuous protocol (Casajús et al., 2001; Castagna et al., 2008; Krustup et al., 2006; Nassis et al., 2010). Castagna et al. (2010) compared the Intermittent Shuttle-Running Test (20-m) with the lactate threshold evaluated for the treadmill (4-minute stages at 8, 10, 12, 14 km/h). Although the authors found that speed at lactate threshold during the intermittent test and treadmill were significantly related (r = 0.82, p < 0.001), the aerobic capacity reduced by approximately 20% in the Intermittent Shuttle-Running Test when compared to continuous velocities. Even with protocol dependence, the application of short distances to measure aerobic capacity in acyclic sports is important to evaluate and prescribe the training load with precision (Castagna et al., 2008; Krustup et al., 2006).

In this study, the elite basketball players presented a high anaerobic index but a similar aerobic capacity in relation to other elite sports. Leicht (2008) reported that elite basketball players showed a significant physiological exertion during the game and as a consequence, considerable utilization of anaerobic metabolism. Narazaki et al. (2009) and Montgomery et al. (2010) demonstrated high predominance of aerobic metabolism in teams without elite players. This fact may occur due to the reduced number of high technical level athletes, which limits frequent substitutions during the match. In this context, it is possible to speculate that training for elite teams is directed to develop anaerobic power resulting in a high technical level of the team enabling constant substitutions. Our data showed that anaerobic performance determined in elite athletes by the RAST was greater when compared with other studies with intermediate teams or physically active subjects (Balčiūnas et al., 2006; Zagatto et al., 2009). On the other hand, players from elite teams show an average value of aerobic capacity which seems to be sufficient to sustain anaerobic metabolism during a single game and throughout the season. In addition, the players with a higher anaerobic index were those with the highest values of aerobic capacity (correlation between FI absolute, Pmax relative and Pavr relative vs. LacMin velocity).

Contrary to the aerobic variables, we did not find differences in anaerobic variables (Pmax, Pavr, Pmin and FI) determined by mathematical and non-mathematical methods for any group. By the values of Power and FI, the visual inspection may be used to identify the variables of the RAST in practice. In addition, the RAST may be an important inductor of the hyperlactatemia method during the initial phase of LacMin. Zagatto et al. (2009) reported that anaerobic contribution of the RAST was very high exhibiting a significant contribution of glycolytic pathway and as consequence LacPeak of approximately 15 mmol·L^−1^. The results of this study showed a LacPeak of approximately 9 mmol·L^−1^ and these values were sufficient to determine the U-curve during the incremental phase for all players.

The Pearson correlations were higher for the RAST mathematical than the visual method. Visual inspection disregards all set points of the RAST and enables evaluation of isolated values of Pmax, Pmin and FI without rigorous criteria. Thus, the associations between Pmax, Pavr and Pmin calculated by the polynomial fit were higher than visual inspection when correlated with the LacMin variables.

The time, velocity and acceleration obtained by simple equations had different values after 5 sprints in the Guards compared with the Centers. The Center group had significantly decreased velocity and acceleration after ∼175 m when compared to the Guard group. These results suggest that the position of basketball players should be considered when evaluating and prescribing training, which is in line with findings of the Sallet et al.’s (2005) study.

In summary, the LacMin test may be considered a good field test to evaluate specific aerobic capacity on the court in basketball players. Considering the elite players, the results of this study are important to characterize the RAST and LacMin responses as well as to provide reference values for athletes and coaches. The specific bidirectional test of 20 m can be used during the incremental phase of LacMin to assess aerobic capacity. However, the LacMin data interpreted by the visual method was different in relation to mathematical analysis for Forwards and All positions groups. On the other hand, the mathematical analysis for the RAST was not different from the visual method. The RAST is a practical protocol to assess the anaerobic index and excellent hyperlactatemia inductor of the LacMin test.

## Figures and Tables

**Table 1:** Values (mean ± standard error) of Power (relative and absolute) and the Fatigue Index (relative and absolute) obtained by the RAST using Visual Inspection and Polynomial fit Curves (3^rd^ degree)

		All positions (n=26)	Guard (n=7)	Forward (n=11)	Center (n=8)
*Visual Inspection*					
Power (Watts)	Max	900.8 ± 39.1	853.1 ± 59.0	923.0 ± 79.8	912.1 ± 42.0
	Med	700.8 ± 22.7	682.8 ± 49.0	700.3 ± 40.7	717.1 ± 26.4
	Min	515.8 ± 21.5	482.2 ± 27.1	532.2 ± 38.9	522.8 ± 38.6
Power (W/kg/s)	Max	9.5 ± 0.4	10.3 ± 0.6	9.8 ± 0.8	8.5 ± 0.5
	Med	7.4 ± 0.3	8.2 ± 0.6	7.4 ± 0.4	6.7 ± 0.3
	Min	5.4 ± 0.2	5.8 ± 0.3	5.7 ± 0.4	4.8 ± 0.4
Fatigue Index (FI)	W/kg/s	0.12 ± 0.01	0.14 ± 0.02	0.13 ± 0.02	0.11 ± 0.02
	%	41.5 ± 2.5	42.1 ± 4.5	40.6 ± 4.4	42.1 ± 4.3
*Polynomial fit curves (3^rd^)*					
Power (Watts)	Max	860.8 ± 54.3	767.1 ± 72.7	868.8 ± 132.0	927.7 ± 58.5
	Med	691.4 ± 34.5	617.8 ± 55.9	704.6 ± 76.1	737.1 ± 38.8
	Min	516.7 ± 20.3	453.8 ± 31.7	538.1 ± 34.5	545.7 ± 28.4
Power (W/kg/s)	Max	9.2 ± 0.5	9.4 ± 0.7	9.5 ± 1.3	8.7 ± 0.8
	Med	7.4 ± 0.3	7.6 ± 0.6	7.7 ± 0.7	6.9 ± 0.3
	Min	5.5 ± 0.2	5.6 ± 0.4	5.9 ± 0.3	5.05 ± 0.5
Fatigue Index (FI)	W/kg/s	0.11 ± 0.02	0.11 ± 0.02	0.11 ± 0.02	0.11 ± 0.04
	%	38.5 ± 2.6	39.9 ± 5.0	35.1 ± 6.0	40.8 ± 2.6

**Table 2:** Values (mean ± standard error) of Lactate Peak (LacPeak), Lactate Minimum Concentration (LacMin), Lactate Minimum Intensity (LacMin) and **Δ** Lactate (mmol/L)= Peak Lactate –Lactate Minimum obtained by Visual Inspection and Polynomial fit Curves (2^rd^ degree)

	All positions (n=26)	Guard (n=7)	Forward (n=11)	Center (n=8)
LacPeak (mmol/L)	9.05 ± 0.36	9.29 ± 0.49	8.46 ± 0.61	9.74 ± 0.65
*Visual Inspection*				
LacMin (mmol/L)	5.50 ± 0.46	5.57 ± 0.97	5.04 ± 0.62	6.18 ± 0.95
LacMin (km/h)	8.96 ± 0.11^*^	9.14 ± 0.15	8.90 ± 0.17^*^	8.83 ± 0.27
Δ Lactate (mmol/L)= Peak	3.64 ± 0.32	3.72 ± 0.42	3.86 ± 0.27	3.18 ± 0.31
Lactate –Lactate Minimum				
*Polynomial fit curves (2^nd^)*				
LacMin (mmol/L)	5.11 ± 0.47	5.32 ± 0.56	4.88 ± 0.43	5.26 ± 0.51
LacMin (km/h)	9.34 ± 0.51	9.41 ± 0.30	9.34 ± 0.60	9.28 ± 0.61
Δ Lactate (mmol/L)= Peak	4.06 ± 0.33	3.96 ± 0.48	4.03 ± 0.29	4.21 ± 0.24
Lactate –Lactate Minimum				
R^2^	0.92 ± 0.01	0.88 ± 0.02	0.93 ± 0.01	0.96 ± 0.01

^*^ Different in relation to Polynomial fit curves (2^nd^ degree) in the same group
